# A digital workflow for design and fabrication of bespoke orthoses using 3D scanning and 3D printing, a patient-based case study

**DOI:** 10.1038/s41598-020-63937-1

**Published:** 2020-04-27

**Authors:** Luke Hale, Emma Linley, Deepak M. Kalaskar

**Affiliations:** 1 UCL Institute of Musculoskeletal Sciences (IOMS), Division of Surgery and Interventional Science, Royal National Orthopaedic Hospital-NHS Trust, Stanmore, Middlesex HA7 4LP United Kingdom; 2 London Spinal Cord Injury Centre, Royal National Orthopaedic Hospital NHS Trust, Stanmore, Middlesex HA7 4LP United Kingdom

**Keywords:** Translational research, Biomimetics, Rehabilitation, Biomedical engineering

## Abstract

This study demonstrates the development and application of a novel workflow for designing and fabricating orthoses, using a combination of 3D scanning and 3D printing technologies. The workflow is applied to a clinically relevant translational case study in a patient with a neurological disorder and complex clinical needs. All traditional and commercial approaches to helping the patient’s cervical instability and resulting ‘head-drop’ had previously failed, with associated progressive deterioration in the patient’s clinical state and posture. The workflow was developed to design and fabricate a bespoke device for this patient with no viable alternative therapy. The workflow was developed to generate 3D printable geometry from obtained 3D scan data. The workflow includes algorithms to relax geometry, distribute material efficiently and for variational cutting of orthosis padding material. The 3D patient scan was validated against actual measurements to ensure accuracy of measurements. A total of four prototypes were produced with each iteration being improved based on patient and clinical feedback. There was a progressive improvement in subjective feedback through each iteration at sites of discomfort and overall comfort score. There was a marked improvement in the patient’s posture with correction at the cervical and lumbar spine with the 3D-printed padded collar being worn for 4 hour periods. This study has implications for the rapid production of personalised orthoses which can help reduce patient waiting time, improve patient compliance, reduce pain and reduce further deterioration. The workflow could form the basis for an integrated process, whereby a single hospital visit results in a bespoke orthosis optimised and personalised for each patient.

## Introduction

Orthoses are externally applied devices that provide structural or functional adjustment to the body. They are intended to improve the user’s level of function and quality of life by guiding, restricting or supporting movement. Traditionally, custom orthoses are made via a laborious, manual process by skilled clinicians, involving casting, sculpting, and moulding of a thermoplastic and fitting to the body^1,2^. This results in waiting times of up to 6 weeks, with multiple patient visits, at a social and economic cost, and no guarantee of patient concordance. Orthotics may have to be revised regularly, requiring the orthosis to be re-made and repetition of this time-consuming and skilled process. The application and combination of new technologies may help to overcome the limitations of these traditional approaches while replicating or improving the mechanical, functional and aesthetic properties.

### 3D printing of orthotic devices

Additive manufacturing, or 3D printing, is a process whereby material is sequentially added to print a 3D object from a computer model. It is becoming an accessible and widespread technology, which has demonstrated numerous applications within medicine, from surgical planning^3^, education^4^, prostheses^5,6^ and drug delivery^7^. 3D printed orthotic devices can be made to have comparable biomechanical properties to traditionally manufactured devices, with potential for fine control over these properties^8–11^. Orthotic designs made via computer-aided design (CAD), can be widely distributed through large open-source communities and then customised by the end-user^12^, allowing adjustment of the resulting model to meet the user needs. This contrasts with manufactured, commercial devices - a ‘one-size-fits-all’ approach - and greatly reduces the cost associated with creating customised, bespoke devices. Patient feedback can be obtained on CAD designs before manufacture, with patient feedback influencing the design process, leading to greater patient involvement and sense of autonomy, thereby hopefully improving compliance^13^.

### 3D scanning in healthcare

3D scanning is a process whereby shape, and sometimes colour, information is collected from a physical object to construct a digital 3D model. In medicine, 3D scanning can be used to rapidly create accurate geometry of the surface of the human body. This has shown wide applicability in healthcare - estimation of burn surface area^14^; anthropometric measurements in scoliosis^15^; monitoring of chronic wounds^16^; and in aesthetic surgery and dermatology^17^. When combined with 3D printing, 3D scanning will likely become an invaluable tool in constructing bespoke orthoses, prostheses and exoskeletons^18,19^. To be applicable in clinical practice, standardised protocols must be developed that enable reliable and reproducible acquisition of scan data^20^.

### Digital workflow for customised design

The development of digital workflows typically involves generating designs based on imaging or 3D scanning data, using procedural or parametric software to create a customised, semi-automated process. Such workflows offer the potential for rapid iteration and generation of prototypes, as well as for non-expert users to generate designs following identification of anatomical landmarks^21^. They have been used to rapidly generate 3D printable geometry for wrist splints from 3D scans^22^, ankle-foot orthoses^10^ and dental implants^23^.

Biomimetics involves replicating Nature’s evolved, well-adapted systems to help solve human problems. By imitating the complex spatial heterogeneity and hierarchical structures present in natural tissues one can create novel, useful materials^24^, anisotropy^25^, or local spatial variation in properties^26,27^. By combining computer models of bone with additive manufacturing technologies, one can create complex scaffolds that mimic both the bone material and internal structure^28^. This study seeks to apply similar principles to the development of a real-world orthosis.

### Existing cervical orthoses

Cervical collars are used for three functions: to stabilise or immobilise the neck following trauma or surgery; to support the neck in cases of chronic neck pain; or to hold the head up in neuromuscular weakness. Most commercial collars are intended for use in trauma; long-term use of these collars is uncomfortable, restrictive and poorly tolerated^29^. Recently, customisable and configurable devices specifically intended for patients with progressive neuromuscular neck weakness have been developed^30^. Detachable support modules facilitate planes of movement, supporting the head whilst not unnecessarily restricting movement, ensuring muscle tone^31^. Such devices offer a degree of customisability by the end user, a step between mass produced commercial devices and bespoke orthotics. However, these collars still rely on accommodating a normalised anatomy, and may not be suitable for patients with unique clinical needs or atypical anatomy, for whom fully bespoke devices may be the only option.

This study demonstrates the development and application of a novel workflow for creating cervical orthoses, using a combination of 3D scanning, biomimetic design and additive manufacturing. The process is validated with a translational case study demonstrating the successful generation of a bespoke device for a patient in whom traditional cervical orthotic approaches had failed.

## Materials and Methods

### Clinical case details

Transverse myelitis is an acute or subacute inflammation of the spinal cord resulting in neurological deficits such as weakness, sensory loss or autonomic dysfunction. The aetiology may be autoimmune or infectious, though in many cases is unknown, and deemed idiopathic. This case study considers the case of a 55-year-old female patient at the time of injury, who became unwell in November 2011 and experienced a 2-month progressive history of bilateral upper limb weakness, neuropathic pain and sensory disturbance. MRI investigations suggested a C4–6 Transverse Myelitits resulting in a central cord syndrome. The patient underwent extensive investigations that identified central and peripheral demyelination. Formal assessment categorized a spinal cord injury at C1, ASIA Impairment Scale (AIS) D Incomplete tetraplegia (High Central Cord Syndrome). The resulting neurological deficits at the time of injury were: significant weakness in her neck muscles so that she was unable to maintain upright neck alignment with a tendency to hold her neck in right side and forward flexion; neck posture that contributed to a flexed/kyphotic upper thoracic spine with poor shoulder girdle alignment, bilateral shoulder subluxation with no active upper limb movements. Whilst able to walk independently she required full assistance for all activities of daily living. Upper limb orthoses were required for stability and pain relief; she was unable to tolerate a cervical collar due to a sense of restricted breathing. Over the intervening years, her cervical posture deteriorated, causing pain, reducing her field of vision and affecting the patency of her airway and general respiratory function. In order to be able to mobilise and visualise her surroundings, the patient would compensate by exaggerating her lumbar lordosis to lean posteriorly, resulting in further deviation of her thoracic and lumbar spine. No viable commercial neck support or traditional bespoke collar fabrication was found that could accommodate her marked cervical kyphosis without resulting in partial compression of her airway and the neurovasculature of her neck in conjunction with an inability to tolerate a feeling of pressure across her chest, compounded by partial sensory denervation of her diaphragm.

Informed consent was given by the patient and her family to produce and evaluate a bespoke orthosis and for online publication of 3D scans, images and clinical case details in an open-access scientific journal. Approval was gained from the Innovation Development Committee at the Royal National Orthopaedic Hospital. All methods were carried out in accordance with relevant guidelines and regulations for production of other bespoke cervical devices, by trained clinicians.

### 3D scanning and its calibration

A 3D surface scan was obtained, with the clinical support of the patients head in a corrected position, using the ‘Artec EVA’, a handheld scanner, with the software Artec Studio 12 Professional used to process 3D surface scans. This scanner has previously shown excellent agreement with high-resolution reference scans for torso and spinal imaging^15^. The 3D scan mesh was validated by comparing real-world measurements to scan data, based on distances between anatomical landmarks^20,32^.

### Design process

**Figure 1:**
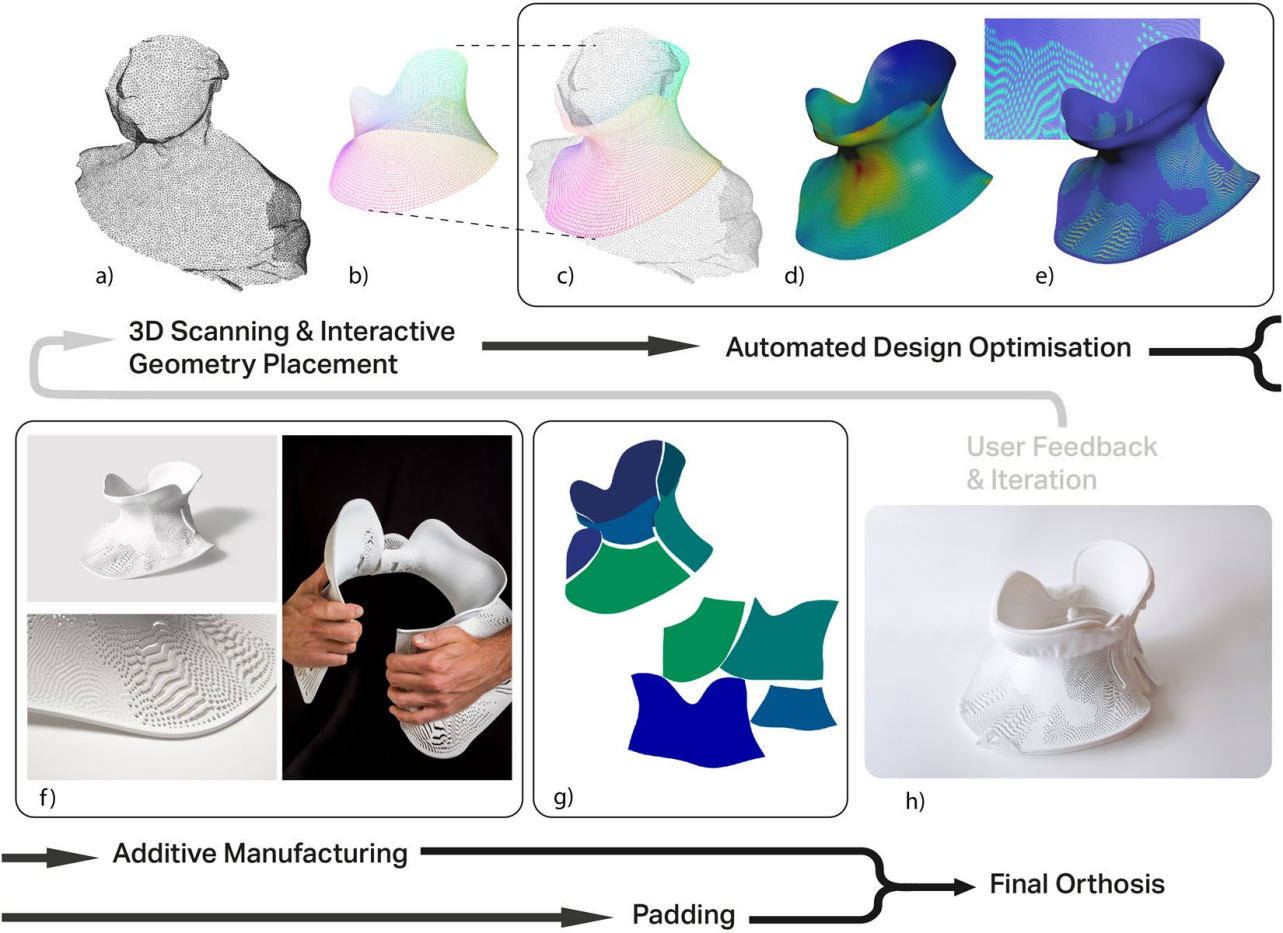
Schematic representation of design workflow and fabrication process of custom orthosis. (**a**) A 3D scan of the patient is acquired using Artec EVA scanner^32^; (**b**) the user positions simple geometry over the region of interest, with feedback on how geometry placement relates to the 3D scan; (**c**) This geometry is fitted then relaxed away from the 3D scan surface to improve patient comfort; (**d**) Deformation energy is mapped to the surface (red = highest deformation energy; blue = lowest deformation energy); (**e**) Deformation energy is used to generate a porous pattern, this porous surface is extruded to form 3D printable geometry. Steps (**c-e**) are automated and require no user input. (**f**) The orthosis is 3D printed using Stratasys Fortus 380mc^32^. (**g**) A variational surface cutting algorithm^33^ is used on the geometry from (**c**) to create a template for cutting orthosis padding. (**h**) The assembly of the printed component and padding results in the final cervical orthosis. The workflow can be repeated and iterated based on feedback from the patient and clinical team. Photos: David Bishop and Matthew Town.

The design process and workflow was developed using Houdini software (SideFX software, version 16.5). The 3D scan mesh is imported into the Houdini software, and the user positions simple guide geometry, a cylinder, over the 3D scan. This geometry is projected onto the 3D scan mesh (Fig. 1a). The projection process uses the point normals of the guide geometry to create ‘rays’ that extend in the direction of the normal. These normals are then reversed, to point inwards towards the 3D scan geometry. If a given ‘ray’ collides with the 3D scan mesh, that point is moved to the collision point on the 3D scan (see supplementary information 3 for video). This creates a mesh that conforms to the 3D scan (Fig. 1b), with easily workable and modifiable geometry. As this projected geometry conforms absolutely to the 3D scan, it is brought away from the body to form a comfortable orthosis (Fig. 1c). This step was achieved by ‘relaxing’ the geometry - the geometry is partially iterated towards the minimal surface solution of same mesh with fixed upper and lower boundaries (see supplementary information 3 for video). The minimal surface is obtained by using a position based dynamics solver in the Houdini software. The upper and lower boundaries of the mesh are fixed in position, whilst all other point positions are moved iteratively by the solver in order to minimise the edge length connecting neighbouring points. This results in a solution for the mesh whereby edge length and area is minimised across the surface, akin to soap bubble formation across two rings. The original projected mesh is moved towards this minimal surface solution until the surface is relaxed away from the patient scan. This mesh is used for a calculation of the deformation energy to control the density of the porous pattern (Fig. 1d).

### Design optimisation

This mesh is used in Houdini’s finite element analysis solver. The mesh is inputted with default stiffness parameters for a solid object; the 3D scan head is modelled with a uniform density of 1 kgm^−3^ acting under gravity on the collar geometry. The internal stresses from this simulation can be stored in the mesh. The larger the deformation, the greater the energy stored in the mesh. The local density of the deformation energy is mapped to the surface, and can be used to influence subsequent steps in the workflow. Specific, physical material parameters were not used, and the mesh was simplified as being isotropic i.e. physical properties of the mesh were modelled as uniform in all dimensions. It should be noted fused deposition modelling (FDM) results in an anisotropic structure, with strength varying according to orientation of extruded filament deposition. This anisotropy could be accounted for in a more accurate simulation, with orientation of deposited material being optimised according to the direction of load.

The porous pattern is generated to improve ventilation, as well as reducing the material cost and weight of the final orthosis. This is achieved using a reaction diffusion algorithm, used to simulate a variety of patterns in nature, with the deformation energy used as input for pattern generation (Fig. 1e; see also supplementary information 2 and 3). There is an inverse relationship between deformation energy and porosity, i.e. at sites of high energy and deformation the structure is less porous. After these pores are created, the flat mesh is split into two parts and the edges are everted to prevent digging into the body. Finally, the mesh is extruded to the required thickness (3 mm) and is ready for 3D printing.

### 3D printing

The prototypes were printed on a Stratasys Fortus 380mc^33^ with a build resolution of 0.178 mm (Fig. 1f). The first hinged prototype was printed in Nylon 12; thereafter, two-part designs were printed in Acrylic Styrene Acrylonitrile (ASA), a UV stable thermoplastic.

### Customised padding

The 3D printed orthosis required padding on internal surfaces for patient comfort. A commercially available cushioning material was bought from Algeos (UK) and used for padding. The fitted and relaxed collar geometry was used as input for a variational surface cutting algorithm (Fig. 1g), which seeks to map a curved surface onto a flat plane with minimal distortion, via optimised cuts of the surface^34^. These optimised cuts divided the collar geometry into flat sections, which were then used as templates for cutting the padding material. Fitting this padding to the 3D printed orthosis produced the wearable collar (Fig. 1h).

### Data acquisition methods

The collar was evaluated by the patient using a modified questionnaire, previously validated for commercial cervical orthoses^29^. The patient evaluated the collar at home, with regular, real-world use. The collar was fitted by a career.

### Statistical analysis

Mean and standard deviation of the maximum errors were calculated for both data acquisition methods. An independent t-test (p < 0.05, 95% CI) was used to compare average maximum errors of both data acquisition methods.

## Results and Discussion

### Patient scanning and accuracy measurements

The Artec EVA 3D scanner has a 3D point accuracy of 0.1 mm^32^. This scanner has previously shown excellent agreement with high-resolution reference scans for torso and spinal imaging^15^. It has previously been validated for acquisition of 3D scan data for bespoke 3D printed devices^18^.

### Design of patient-specific orthosis

This process was integrative and relied on close communication between engineering, the clinical team and the patient. A total of 4 prototypes were designed to capture and rectify patient and clinical feedback. With each design, patient comfort and usability was improved by making small changes to adopt design considerations and to correct cervical posture and improve patient compliance.

The first prototype validated the 3D scanning to 3D printing workflow - i.e. to successfully take a 3D scan and create 3D printable geometry that conforms to the patient. The first design was hinged along one side, and printed with a semi-flexible nylon material. Although this design offered the possibility of an easily fitted, wrap-around ‘clamshell’ structure, the material was not sufficiently flexible to be easily opened to accommodate the neck. This design was separated along the hinge and fitted to the patient. This collar was poorly tolerated as it was too close fitting and could not accommodate the necessary padding.

The second prototype involved a number of modifications - the edges of the collar were everted, away from the skin to prevent skin pressure at the collar edges. The clearance between the skin and the orthotic surface was increased to accommodate padding. The collar was printed in two opposing sections, with straps at the sides. The third prototype involved the manual removal of material away from the second prototype, at the shoulders and head, and a corrective adjustment to the patient’s tendency of lateral and axial rotation of the head with padding. The fourth prototype was re-printed taking into account previous improvements, including the removal of material at sites of discomfort from prototypes 2 and 3.

The patient-specific cervical collar was designed and produced using the workflow described in section 2.2. The final prototype took 2 min 45 sec to generate 3D printable geometry from 3D scan data (Intel Core i7 8700 K 3.70 GHz), i.e. this is the time taken to proceed through the procedural workflow. The FEM process combined with the porosity algorithm reduced the volume of the orthosis by 13%, with the added benefit of improving ventilation. A FEM approach with topological optimisation may mean the material is used more efficiently, although such methods do not factor in patient comfort or aesthetics. The resulting 3D printed orthosis was provided to the clinical team for inspection and discussion. Once a design was finalised, it was 3D printed and fitted to the patient by an Occupational Therapist (the collar was fitted at home by the carer).

### Fabrication of bespoke collar

Final fabrication of orthoses was done using Stratasys Fortus 380mc 3D printer. Acrylic Styrene Acrylonitrile (ASA) was identified as the most appropriate material with the required mechanical properties and surface finish required for the orthosis in terms of strength and comfort. The total time for printing and post-processing of the 3D printed collar was 48 hours (~2 days).

The collar was fitted with commercial orthotic padding for durability and patient comfort. The variational surface cutting algorithm ensured the padding conformed to both the patient’s body and the inner surface of the orthosis and automated the process of creating a template from a 2D plane that conformed to the 3D orthosis with minimal distortion. Total time from scanning to delivery of 3D printed orthosis was approximately 72 hrs (3 days). The time taken for supplying traditional orthoses typically involves separate patient appointments for initial assessment, measurement, evaluation, adjustment and final supply of the device, with each appointment lasting around 40 minutes in a complex case^2^. Manufacturing is performed by a skilled technician in between these appointments. For a typical orthosis, this results in a turnaround time of around 6 weeks in our hospital. With the use of a 3D scanning process, it is possible capture all required measurements of patients in short appointments lasting 10–15 min, removing the need for moulding or casting steps, and saving considerably on labour-hours at busy orthotics units. We anticipate by having integrated 3D design, printing facilities within orthotic divisions of hospitals, the delivery time for 3D printed orthosis can be further reduced in future. CAD/CNC controlled cutting of the padding material would enable the padding process to be fully automated, and integrated into the design workflow in future.

### Clinical case study

**Figure 2:**
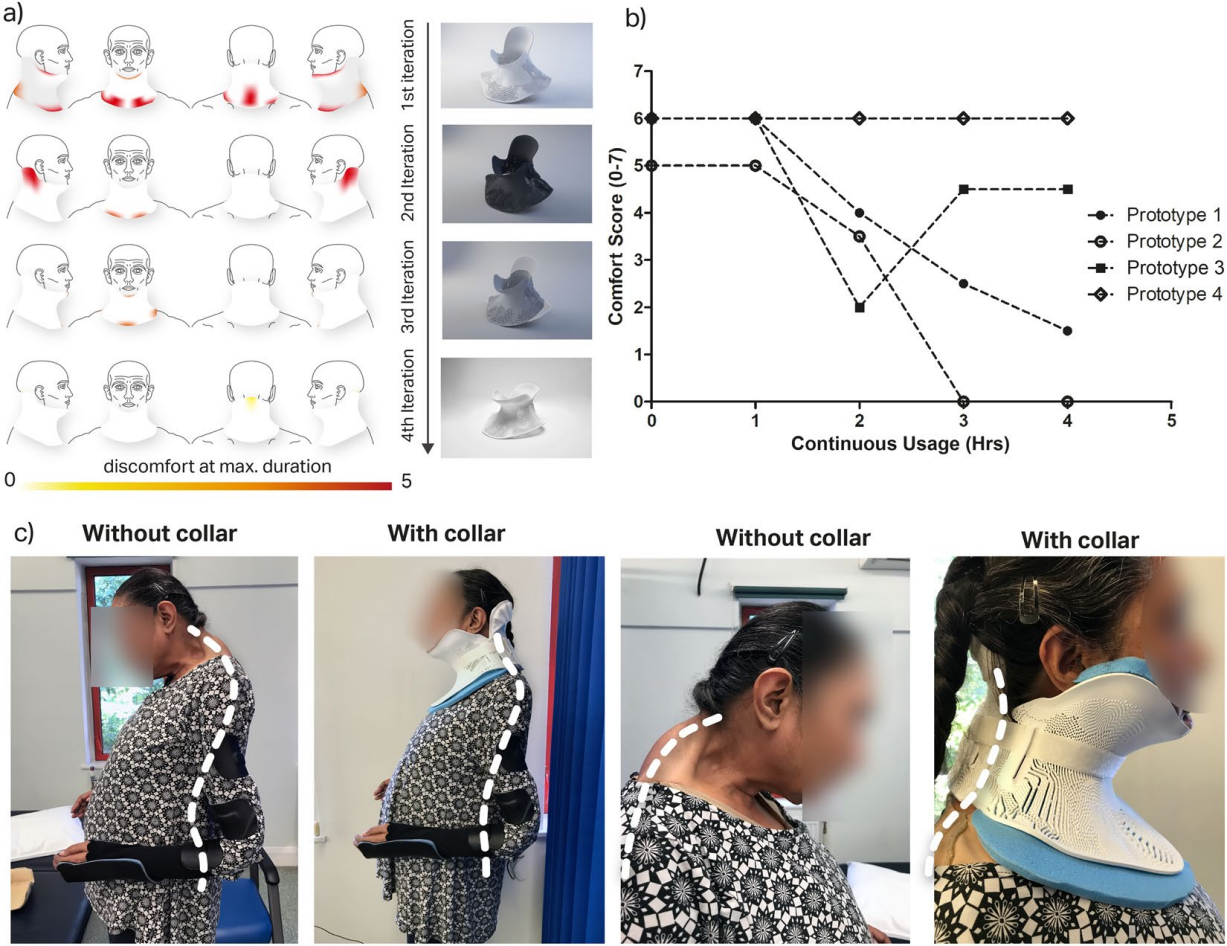
(**a**) Red area showing reported sites of discomfort, at maximum duration with different collar iterations. (scale: 0 = no reported discomfort, 5 = severe discomfort); (**b**) showing patient self-reported comfort over time with continuous use, over different collar iterations; (**c**) Photographs of patient wearing 4th prototype comparison of posture and spine position pre and post application of orthosis shows spine position with and without collar with noted improvement of head position, significant reduction of cervical kyphosis and reduction in the pronounced, compensatory lordosis at the lumbar spine.

Four prototypes were produced for this case study, with each prototype being altered based on patient feedback from the previous iteration. The patient and clinical team was involved throughout the design process, improving patient compliance and engagement. Each prototype was evaluated using a modified questionnaire previously used to evaluate commercial cervical orthoses (supplementary information 1). There was a progressive improvement in subjective feedback through each iteration as represented by the site of discomfort and comfort score shown in Fig. 2a,b; the collar could be tolerated for progressively longer with greater levels of comfort. There was a marked improvement in posture whilst wearing the collar, with reduction of the compensatory exaggerated lordosis at the lumbar spine (Fig. 2c). The neutral head position reduced the patients reported pain, enabled a wider field of vision, eased ability to swallow, reduced lumbar lordosis and backward lean thereby improving stability in static standing and mobility. The application of the variational surface cutting algorithm provided custom fit cutting of orthosis padding, which resulted in a notable improvement in patient comfort and compliance as shown for prototype 3 (manually cut padding) vs prototype 4 (cut using algorithm) (Fig. 2a). The final (fourth) prototype is tolerated for 4 hours, with gradual increase in usage time. The workflow was therefore validated in producing clinically-beneficial bespoke orthoses whilst also facilitating iteration, modification and improvement of the design.

This study demonstrates the feasibility of a 3D scanning and printing process to design and manufacture a patient specific cervical collar, although the workflow could equally be applied to other parts of the body. Through a collaborative process with the patient and the clinical team, the cervical collar was custom made to the patient’s anatomy, whilst bringing the collar away from the airway and other sensitive structures at the anterior of her neck. The use of 3D scanning obviates the need for poorly tolerated casting and pressure on the chest. This also helped to reduce calling in patient for subsequent iterative modifications saving both cost and travel time for patients.

The process of adjusting based on patient considerations meant the patient felt involved in the design. This had an additional psychological advantage that aided compliance; from having no alternative, the patient now wears the collar around 4 hours per day. Such an approach may therefore have a place in patients with complex clinical needs, where traditional approaches have failed. Furthermore, whereas traditional methods of producing bespoke orthoses require multiple patient visits, which may be economically and practically unfeasible, 3D scanning offers the ability for designs to be reviewed and approved by the patient or clinical team remotely, with the ability to preview the orthoses on the 3D scanned model of the patient. The workflow enables different permutations of designs to be generated with variations of constraints; with selections based on design considerations or preference. There are no sacrificial steps, compared with casting, or formation of waste product. Streamlining this process, with a dedicated 3D printer, could result in a point-of-care 3D printing process, with patient attending hospital, having 3D scan and leaving with orthosis the following day.

### Optimisation of process

The 3D scanning process was complicated in this case by the patient’s clinical condition. More reliable results may be obtained through future optimisation of scanning protocols and techniques in such cases. The use of a procedural workflow enabled rapid generation of 3D printable geometry (<3 minutes). The initial required steps in the workflow could be performed by a clinical team, relying on inputting guide geometry and positioning it over the region of interest on the 3D scan. By far the most time-consuming and time-limiting stage of the process is the 3D printing of the orthosis. However, with integrated 3D printing facilities and access to multiple printers can reduce this time. The cost of the 3D printed collar was £325, with printing undertaken at a commercial 3D printing company with the necessary printing facilities. This compares to an estimate of £350 for a similar device produced using traditional methods at our hospital.

Fused filament fabrication, the most commonly used printing technology, involving deposition of a heated filament through an extruder, is inherently slow. Selective laser sintering (SLS) may be a cheaper and faster process, although is generally less accessible and therefore was not used in this proof-of-concept study. Newer printing technologies such as large-scale rapid liquid printing, or ‘CLIP’ (Continuous liquid interface production of 3D objects) are promising as potentially much more rapid production additive manufacturing technologies^35^. Further optimisation of the design via a more intensive FEM approach may reduce material cost and print time.

## Conclusion

This study demonstrated the combination of 3D scanning and 3D printing in a clinically relevant and validated case study. The workflow was demonstrated to be feasible and potentially more rapid than traditional methods, with quantifiable clinical and patient benefits. In future work, the proposed workflow will be extended to larger group of patients with varying anatomy and cervical pathology, in whom traditional or commercial approaches have failed This study has implications for the rapid production of personalised orthoses which can help reduce patient waiting time, improve patient compliance, reduce pain and reduce further deterioration. The workflow could form the basis for an integrated process, whereby a single hospital visit results in a bespoke orthosis optimised and personalised for each patient.

## Supplementary information

Supplementary information 1.

Supplementary information 2.

Supplementary information 3.

Supplementary information 4.

Supplementary information 5.

Supplementary information 6.

## References

[1] International Committee of the Red Cross. Manufacturing guidelines: Lower Limb and Upper Limb Orthoses, 2006.

[2] British Association of Prosthetists and Orthotists. Standards for best practice. Paisley: British Association of Prosthetists and Orthotists, 2013.

[3] Ahmed H (2018). From Improved Diagnostics to Presurgical Planning: High-Resolution Functionally Graded Multimaterial 3D Printing of Biomedical Tomographic Data Sets. 3D Print. Addit Manuf.

[4] Garcia J, Yang Z, Mongrain R, Leask RL, Lachapelle K (2018). 3D printing materials and their use in medical education: a review of current technology and trends for the future. BMJ simulation & technology enhanced learning.

[5] Chen RK, Jin Y-a, Wensman J, Shih A (2016). Additive manufacturing of custom orthoses and prostheses—A review. Additive Manufacturing.

[6] Sengeh DM, Moerman KM, Petron A, Herr H (2016). Multi-material 3-D viscoelastic model of a transtibial residuum from *in-vivo* indentation and MRI data. Journal of the Mechanical Behavior of Biomedical Materials.

[7] Muwaffak Z (2017). Patient-specific 3D scanned and 3D printed antimicrobial polycaprolactone wound dressings. International journal of pharmaceutics.

[8] Dombroski CE, Balsdon ME, Froats A (2014). The use of a low cost 3D scanning and printing tool in the manufacture of custom-made foot orthoses: a preliminary study. BMC research notes.

[9] Wojciechowski E (2019). Feasibility of designing, manufacturing and delivering 3D printed ankle-foot orthoses: a systematic review. Journal of Foot and Ankle Research.

[10] Cha YH (2017). Ankle-Foot Orthosis Made by 3D Printing Technique and Automated Design Software. Applied Bionics and Biomechanics.

[11] Portnova AA, Mukherjee G, Peters KM, Yamane A, Steele KM (2018). Design of a 3D-printed, open-source wrist-driven orthosis for individuals with spinal cord injury. PloS one.

[12] E-NABLE. *Enabling the Future* [Online]. Available: http://enablingthefuture.org [Accessed 02/12/2019]

[13] Wong MS (2011). Computer-aided design and computer-aided manufacture (CAD/CAM) system for construction of spinal orthosis for patients with adolescent idiopathic scoliosis. Physiotherapy theory and practice.

[14] Retrouvey H, Chan J, Shahrokhi S (2018). Comparison of two-dimensional methods versus three-dimensional scanning systems in the assessment of total body surface area estimation in burn patients. Burns: journal of the International Society for Burn Injuries.

[15] Grant CA, Johnston M, Adam CJ, Little JP (2019). Accuracy of 3D surface scanners for clinical torso and spinal deformity assessment. Medical engineering & physics.

[16] Farrar E, Pujji O, Jeffery S (2017). Three-dimensional wound mapping software compared to expert opinion in determining wound area. Burns: journal of the International Society for Burn Injuries.

[17] Arenas M (2017). Individualized 3D scanning and printing for non-melanoma skin cancer brachytherapy: a financial study for its integration into clinical workflow. Journal of contemporary brachytherapy.

[18] Ben Abdallah I., Bouteraa Y., Rekik C. Design and development of 3d printed myoelectric robotic exoskeleton for hand rehabilitation (2017).

[19] Cui L, Phan A, Allison G. Design and fabrication of a three dimensional printable non-assembly articulated hand exoskeleton for rehabilitation. In: 2015 37th Annual International Conference of the IEEE Engineering in Medicine and Biology Society (EMBC)) (2015). 10.1109/EMBC.2015.7319425 26737325

[20] Geierlehner A., Malferrari S., Kalaskar D.M. The optimization of a 3D scanning technique applied for 3D printing of bespoke medical devices. Journal of 3D Printing in Medicine, (2019).

[21] Li J, Tanaka H (2018). Feasibility study applying a parametric model as the design generator for 3D-printed orthosis for fracture immobilization. 3D Print Med.

[22] Li J, Tanaka H (2018). Rapid customization system for 3D-printed splint using programmable modeling technique - a practical approach. 3D printing in medicine.

[23] Surovas A (2019). A digital workflow for modeling of custom dental implants. 3D Print Med.

[24] Liu Z, Meyers MA, Zhang Z, Ritchie RO (2017). Functional gradients and heterogeneities in biological materials: Design principles, functions, and bioinspired applications. Progress in Materials Science.

[25] Jon (2016). Procedural voronoi foams for additive manufacturing. ACM Trans Graph.

[26] Doubrovski EL (2015). Voxel-based fabrication through material property mapping: A design method for bitmap printing. Computer-Aided Design.

[27] Li Y-C, Zhang YS, Akpek A, Shin SR, Khademhosseini A (2016). 4D bioprinting: the next-generation technology for biofabrication enabled by stimuli-responsive materials. Biofabrication.

[28] Gomez S, Vlad MD, Lopez J, Fernandez E (2016). Design and properties of 3D scaffolds for bone tissue engineering. Acta biomaterialia.

[29] Langley J (2018). A comfort assessment of existing cervical orthoses. Ergonomics.

[30] Pancani S (2016). Assessment of the Sheffield Support Snood, an innovative cervical orthosis designed for people affected by neck muscle weakness. Clinical biomechanics (Bristol, Avon).

[31] Pancani S, Tindale W, Shaw PJ, Mazzà C, McDermott CJ (2018). Efficacy of the Head Up collar in facilitating functional head movements in patients with Amyotrophic Lateral Sclerosis. Clinical Biomechanics.

[32] Artec3d. Artec Eva specifications. (2018). User Guide Artec Studio 12 (2017).

[33] Sharp N, Crane K (2018). Variational Surface Cutting. ACM Trans Graph.

[34] Stratasys. Stratasys Fortus 380mc K5 Series User Guide (2015).

[35] Tumbleston JR (2015). Continuous liquid interface production of 3D objects. Science..

