# Supplementary figures and images for: A digital workflow for design and fabrication of bespoke orthoses using 3D scanning and 3D printing, a patient-based case study
Source: Sci Rep. 2020 Apr 27;10:7028. doi: 10.1038/s41598-020-63937-1 (PMC7184736; doi:10.1038/s41598-020-63937-1)

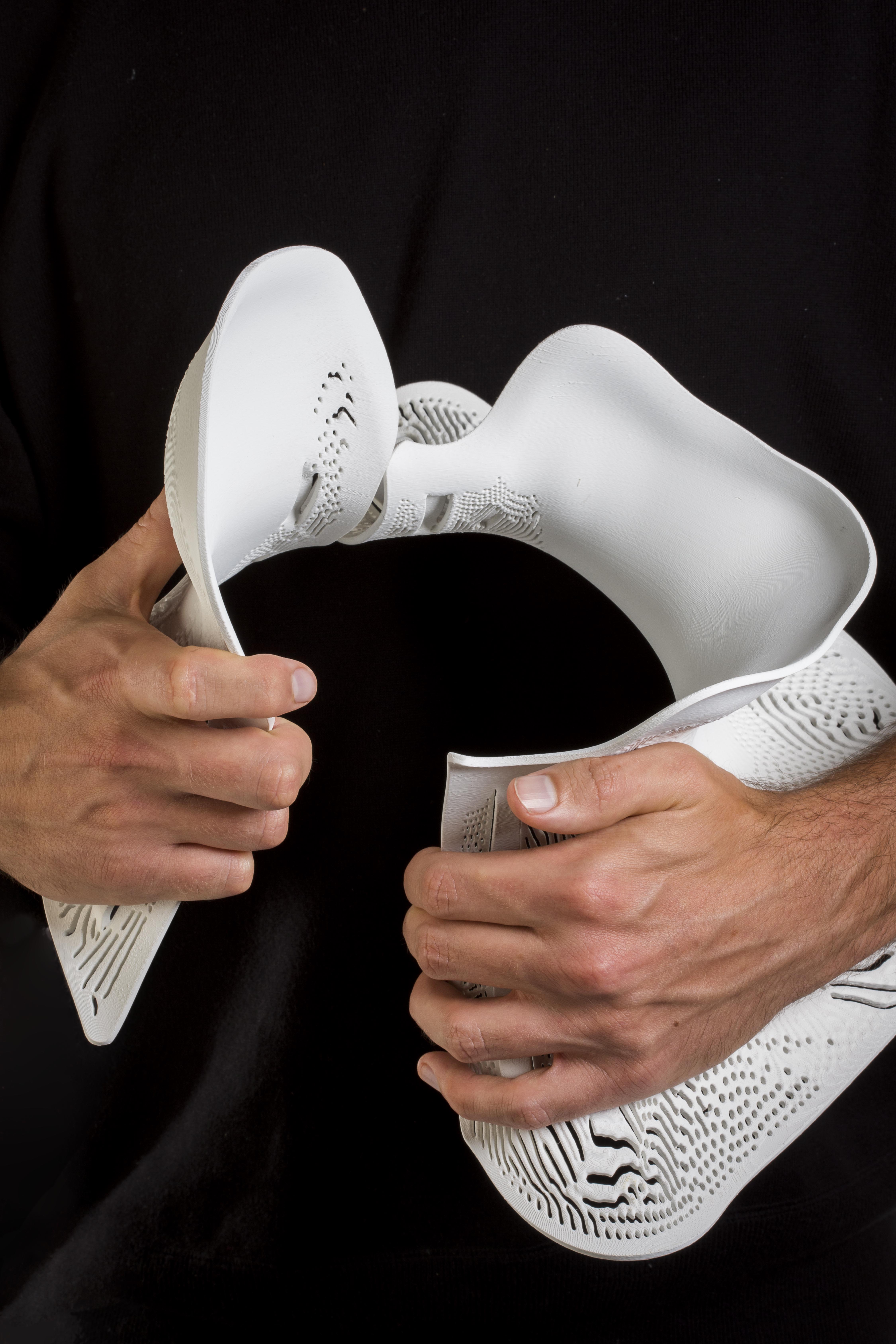

Supplement: Supplementary file 1 — Supplementary information 1. [file 41598_2020_63937_MOESM1_ESM.jpg]

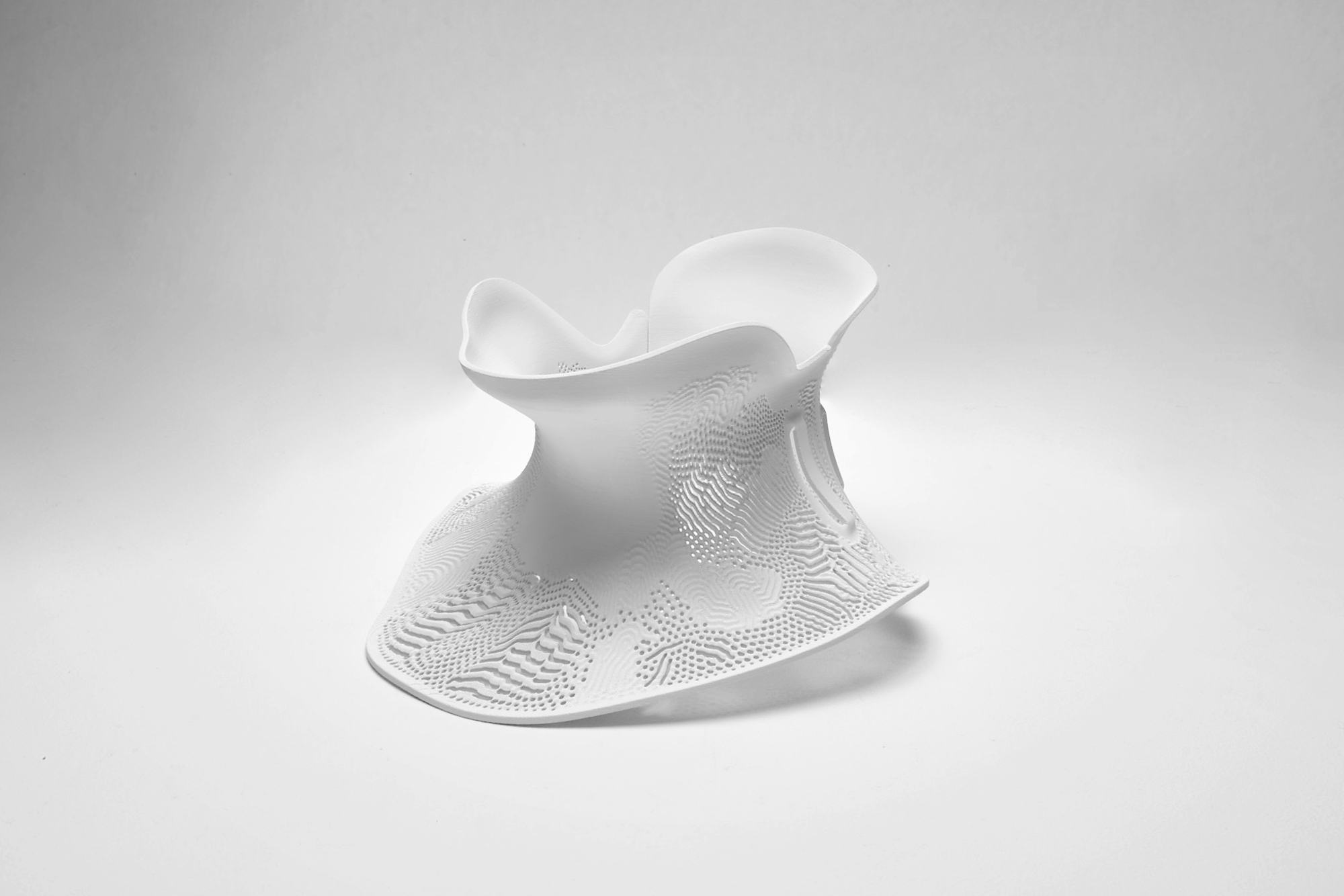

Supplement: Supplementary file 2 — Supplementary information 2. [file 41598_2020_63937_MOESM2_ESM.jpg]

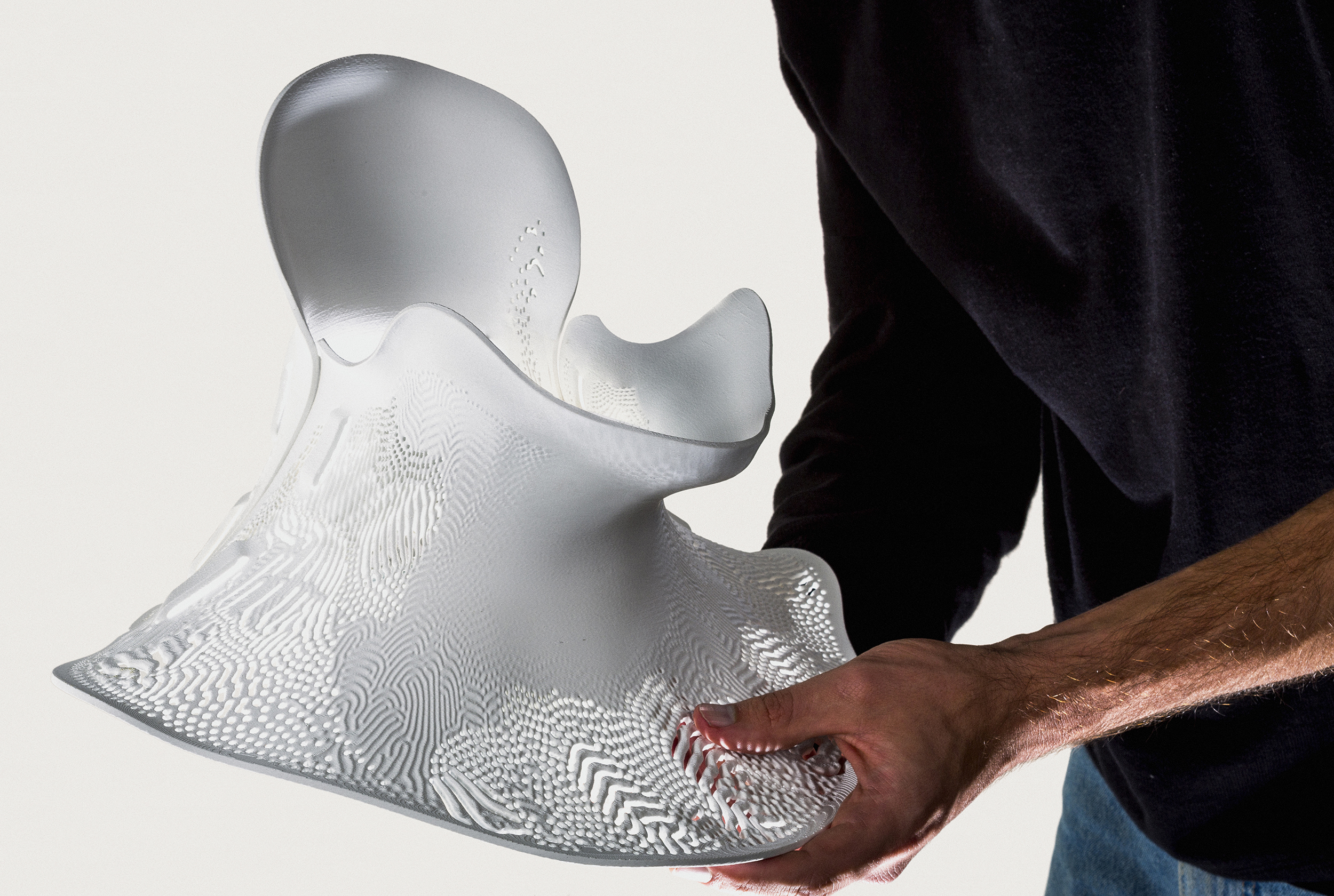

Supplement: Supplementary file 3 — Supplementary information 3. [file 41598_2020_63937_MOESM3_ESM.jpg]
