# Supplementary material for: A digital workflow for design and fabrication of bespoke orthoses using 3D scanning and 3D printing, a patient-based case study
Source: Sci Rep. 2020 Apr 27;10:7028. doi: 10.1038/s41598-020-63937-1 (PMC7184736; doi:10.1038/s41598-020-63937-1)
Supplement: Supplementary file 4 — Supplementary information 4. [file 41598_2020_63937_MOESM4_ESM.docx]

**INSTRUCTIONS**

There are two sections in this questionnaire;

- - Section A requires completion at intervals.
  - Section B should be completed at the end of the test

Assessment of Cervical Collar

Post-modification (3rd iteration)

On the following page there are four sets of images, identical to the example pictured below. Please could you circle on the images the areas you felt discomfort during the specified periods, and also indicate the severity of discomfort on the scale below the images.


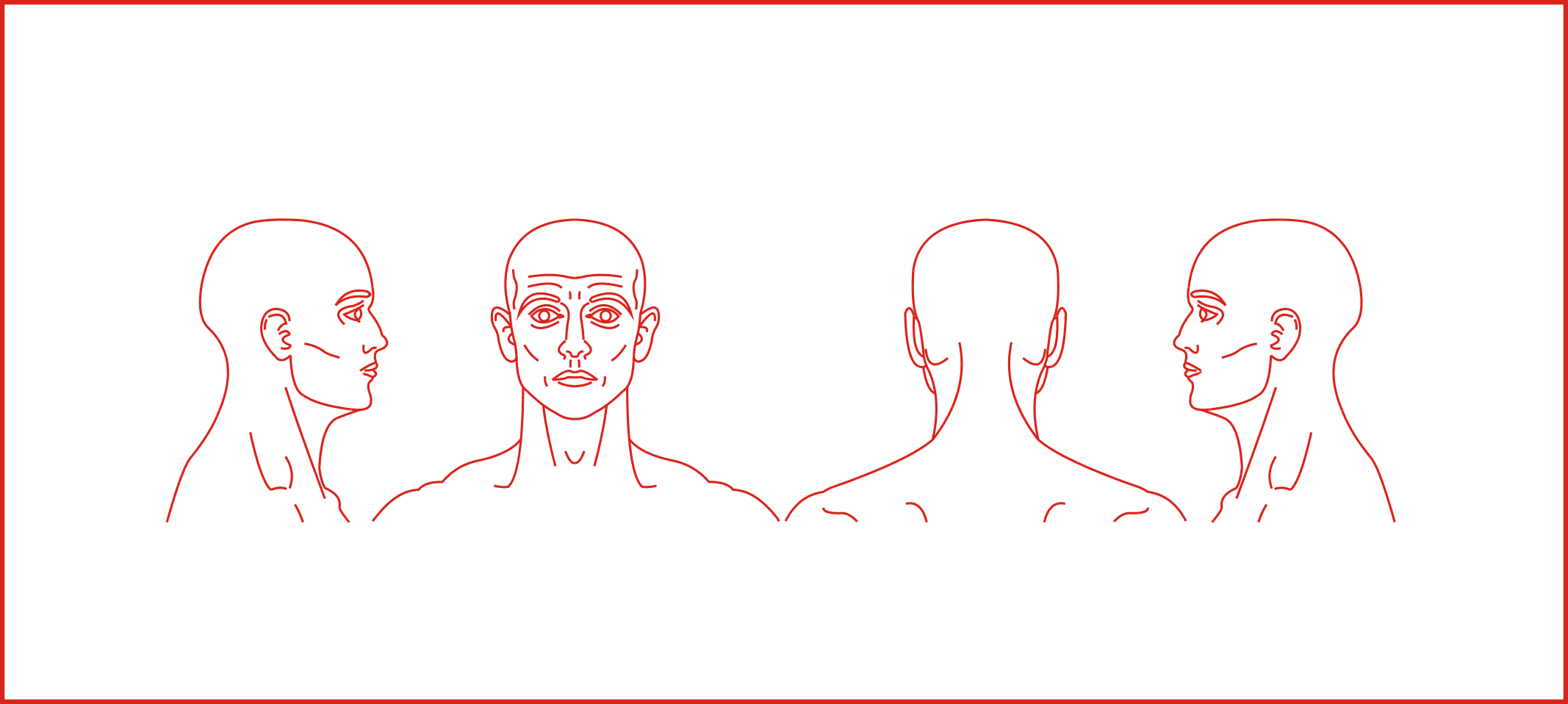


Example image.

✓

No

discomfort

Severe

Pain

A little

painful

A little

discomfort

Very

uncomfortable

Very

painful

Extremely uncomfortable


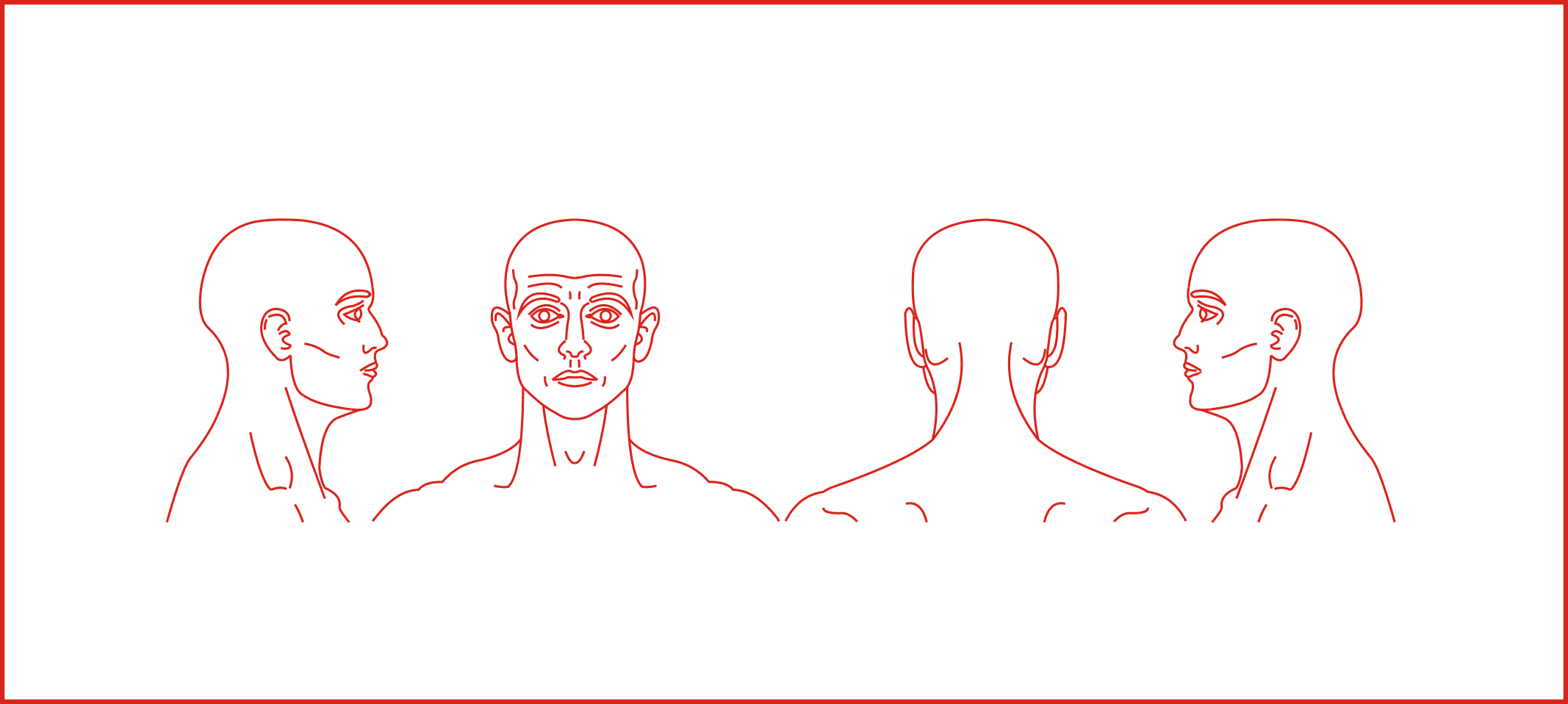


After 4 hours

No

discomfort

Severe

Pain

A little

painful

A little

discomfort

Very

uncomfortable

Very

painful

Extremely uncomfortable


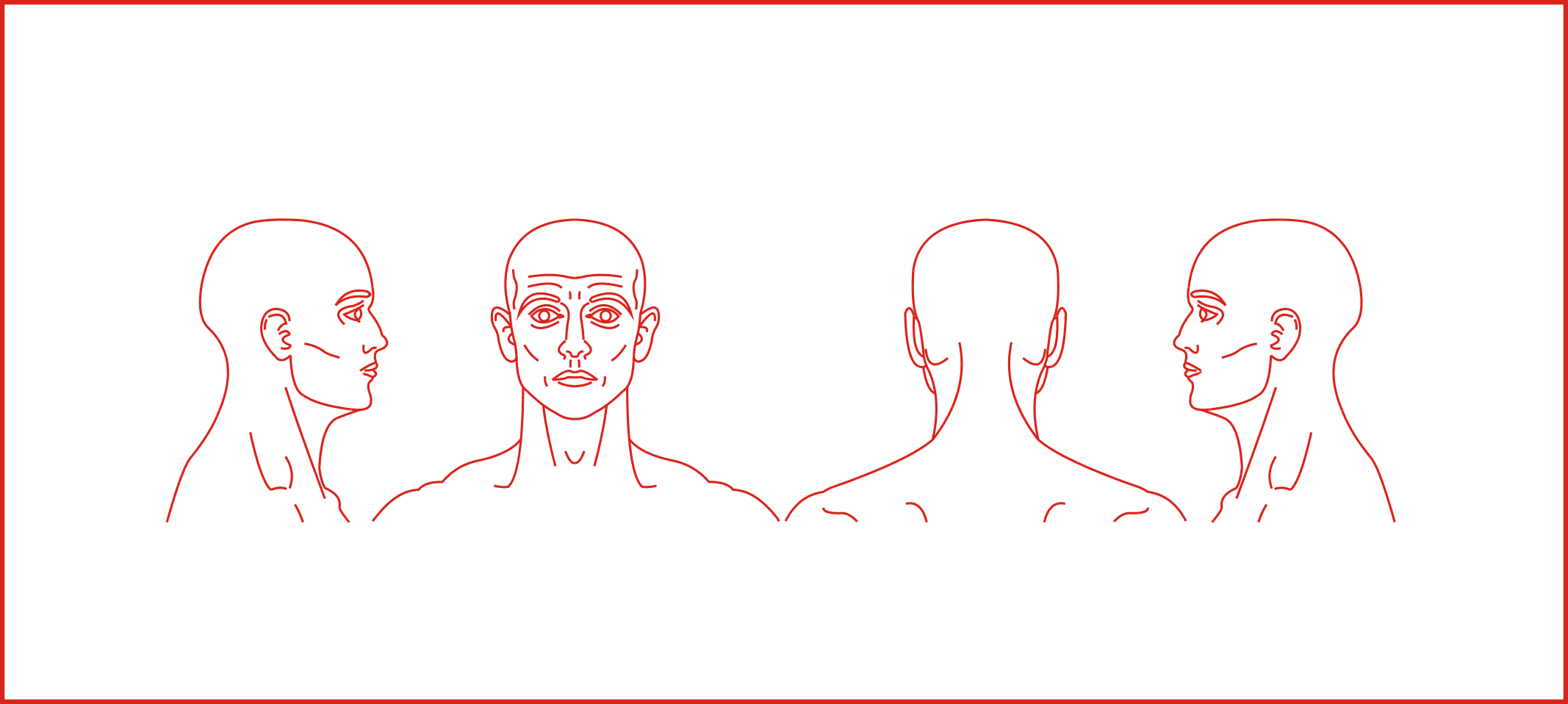


After 3 hours

No

discomfort

Severe

Pain

A little

painful

A little

discomfort

Very

uncomfortable

Very

painful

Extremely uncomfortable


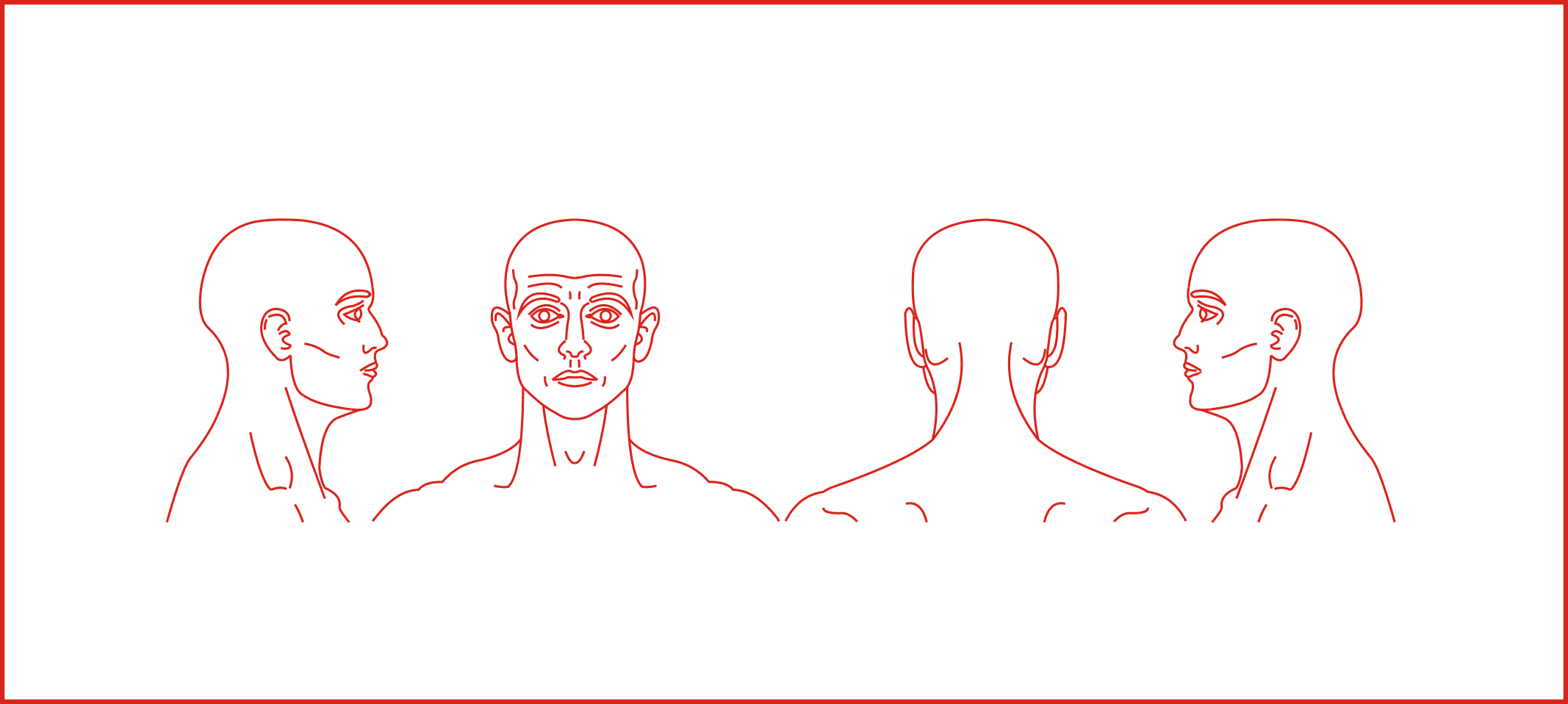


After 2 hours

No

discomfort

Severe

Pain

A little

painful

A little

discomfort

Very

uncomfortable

Very

painful

Extremely uncomfortable


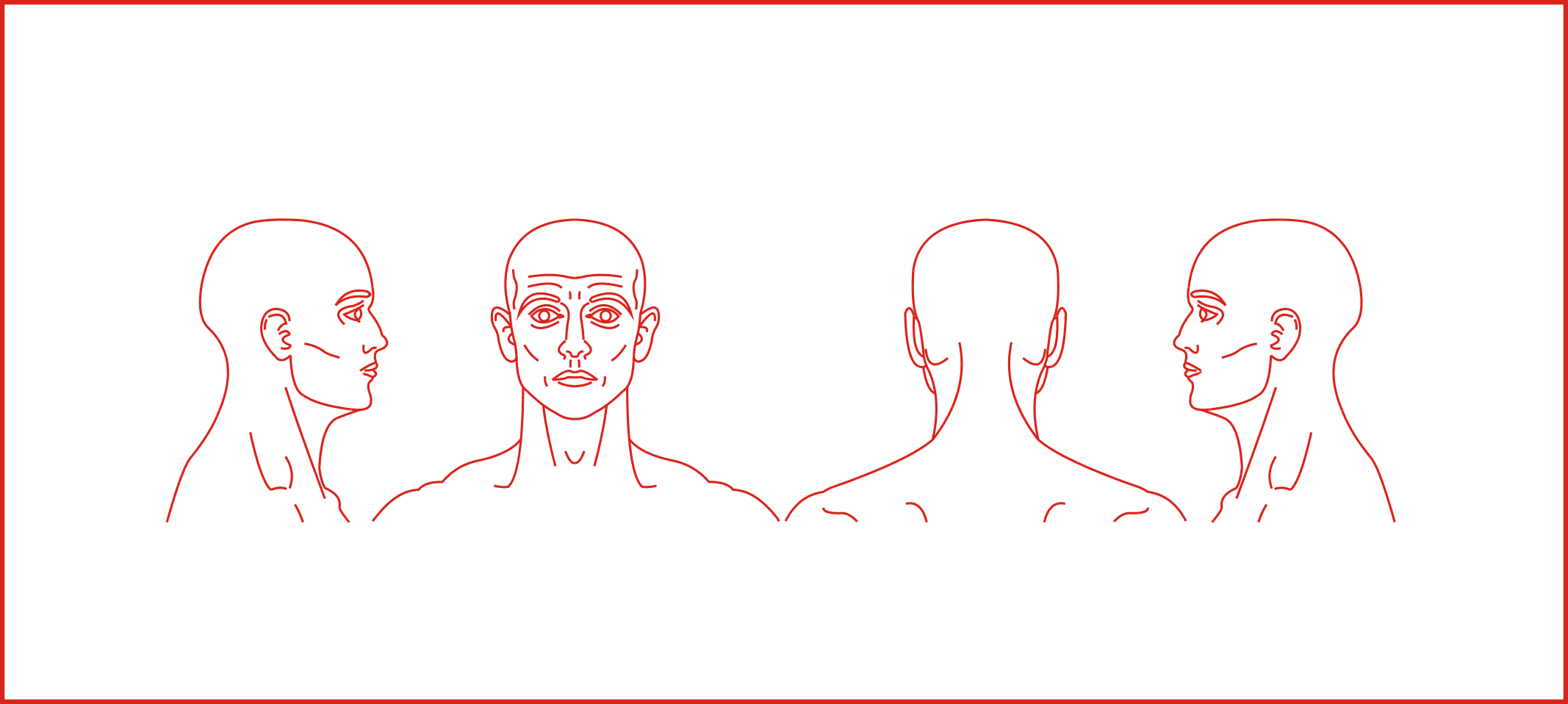


After 1 hour

No

discomfort

Severe

Pain

A little

painful

A little

discomfort

Very

uncomfortable

Very

painful

Extremely uncomfortable

Please fill in this section at one hourly intervals. Indicating your level of comfort and any sites of discomfort.

**SECTION A**

Strongly

agree

Disagree

Agree

Disagree

somewhat

Strongly

disagree

neither

agree or disagree

Agree

somewhat

I found it very hard to breath wearing this collar.

✓

Strongly

agree

Disagree

Agree

Disagree

somewhat

Strongly

disagree

neither

agree or disagree

Agree

somewhat

Strongly

agree

Disagree

Agree

Disagree

somewhat

Strongly

disagree

neither

agree or disagree

Agree

somewhat

Strongly

agree

Disagree

Agree

Disagree

somewhat

Strongly

disagree

neither

agree or disagree

Agree

somewhat

Strongly

agree

Disagree

Agree

Disagree

somewhat

Strongly

disagree

neither

agree or disagree

Agree

somewhat

Strongly

agree

Disagree

Agree

Disagree

somewhat

Strongly

disagree

neither

agree or disagree

Agree

somewhat

Strongly

agree

Disagree

Agree

Disagree

somewhat

Strongly

disagree

neither

agree or disagree

Agree

somewhat

**SECTION B**

Please read the following statements and place a mark on the circle you feel best describes your response, as shown in the example below.

I experienced no problems drinking whilst wearing this collar.

I experienced no difficulties eating whilst wearing this collar.

This collar caused no restriction to my natural breathing.

This collar caused no restriction to my natural swallowing.

I felt that this collar offered support.

I experienced no perspiration around my head, shoulders or neck whilst wearing this collar.

Strongly

agree

Disagree

Agree

Disagree

somewhat

Strongly

disagree

neither

agree or disagree

Agree

somewhat

I find this collar visually attractive.

I felt no frustration at all whilst wearing this collar.

Strongly

agree

Disagree

Agree

Disagree

somewhat

Strongly

disagree

neither

agree or disagree

Agree

somewhat

Please feel free to write any other comments or observations you have regarding your experience of wearing the collar.
