# Supplementary material for: A digital workflow for design and fabrication of bespoke orthoses using 3D scanning and 3D printing, a patient-based case study
Source: Sci Rep. 2020 Apr 27;10:7028. doi: 10.1038/s41598-020-63937-1 (PMC7184736; doi:10.1038/s41598-020-63937-1)
Supplement: Supplementary file 5 — Supplementary information 5. [file 41598_2020_63937_MOESM5_ESM.docx]

The porous pattern is achieved using the Gray-Scott algorithm for reaction diffusion, used to simulate a variety of patterns in nature:


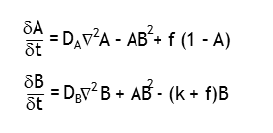


Differential equation defines the change in concentrations of A and B over time. ‘f’ is the feed rate of A; ‘k’ is the ‘kill’ rate of B; dA and dB are the diffusion rates of A and B, respectively. These values are adjusted based on the surrounding concentrations of A or B via a Laplacian Operator (∇2).

By varying the kill and feed rate across the mesh, using the values of the deformation energy obtained through FEM to vary these coefficients, one can achieve a mesh that has variable strength and porosity.
